# Protein Carbonylation and Heat Shock Proteins in Human Skeletal Muscle: Relationships to Age and Sarcopenia

**DOI:** 10.1093/gerona/glu007

**Published:** 2014-03-12

**Authors:** Maria R. Beltran Valls, Daniel J. Wilkinson, Marco V. Narici, Kenneth Smith, Bethan E. Phillips, Daniela Caporossi, Philip J. Atherton

**Affiliations:** ^1^Department of Movement, Human and Health Sciences, Unit of Biology, Genetics and Biochemistry, University of Rome “ForoItalico,”Italy.; ^2^Division of Medical Sciences & Graduate Entry Medicine, MRC-ARUK Centre of Excellence for Musculoskeletal Ageing Research, University of Nottingham, Royal Derby Hospital Centre.

**Keywords:** Sarcopenia, Mitochondria, Carbonylation, Heat shock protein.

## Abstract

Aging is associated with a gradual loss of muscle mass termed sarcopenia, which has significant impact on quality-of-life. Because oxidative stress is proposed to negatively impact upon musculoskeletal aging, we investigated links between human aging and markers of oxidative stress, and relationships to muscle mass and strength in young and old nonsarcopenic and sarcopenic adults. Sixteen young and 16 old males (further subdivided into “old” and “old sarcopenic”) were studied. The abundance of protein carbonyl adducts within skeletal muscle sarcoplasmic, myofibrillar, and mitochondrial protein subfractions from musculus vastus lateralis biopsies were determined using Oxyblot immunoblotting techniques. In addition, concentrations of recognized cytoprotective proteins (eg, heat shock proteins [HSP], αβ-crystallin) were also assayed. Aging was associated with increased mitochondrial (but not myofibrillar or sarcoplasmic) protein carbonyl adducts, independently of (stage-I) sarcopenia. Correlation analyses of all subjects revealed that mitochondrial protein carbonyl abundance negatively correlated with muscle strength ([1-repetition maximum], *p* = .02, *r* ^2^ = −.16), but not muscle mass (*p* = .13, *r* ^2^ = −.08). Abundance of cytoprotective proteins, including various HSPs (HSP 27 and 70), were unaffected by aging/sarcopenia. To conclude, these data reveal that mitochondrial protein carbonylation increases moderately with age, and that this increase may impact upon skeletal muscle function, but is not a hallmark of (stage-I) sarcopenia, per se.

Beyond 50 years, skeletal muscle mass declines at 0.8%–0.98%/year in men and 0.64%–0.7%/year in women (1), a process termed sarcopenia (from the Greek “sarx” for flesh and “penia” for loss) by Irwin Rosenberg in 1989 (2). Sarcopenia impacts mobility, limiting independent living and quality-of-life, and can lead to increased risk of cardiovascular and metabolic disease (3). However, although the causes of sarcopenia are multivalent, involving for example, hormonal changes, neurodegeneration, inflammation, oxidative stress (4), and anabolic resistance (5), mechanistic explanations remain poorly defined.

Perhaps the most established theory of aging is The Free Radical Theory of Aging (6), where excessive reactive oxygen species (ROS) damage proteins, lipids, and DNA when not appropriately balanced by cytoprotective (ie, antioxidant) mechanisms (7). Proteins, for instance, can be modified directly or indirectly by ROS and by reaction with secondary products of lipid peroxidation like 4-hydroxy-2-nonenal or malondialdehyde, generating different species of oxidized products (8). Accumulation of oxidized proteins in muscle is thought to induce cellular/tissue dysfunction, and oxidized products may also be resistant to proteases, leading to further cellular damage or dysfunction (9). Accumulation of carbonyl groups (aldehydes and ketones) on protein side chains occur as a result of oxidation and this is considered a robust biochemical marker of oxidative stress (10).

Heat shock proteins (HSPs) are important components of the protective machinery against a multitude of stressors (heat shock, oxidative stress, inflammation, etc.) (11). They are a family of cytoprotective proteins that recognize and prevent the accumulation of damaged protein by repairing/refolding damaged ones or degrading irreversibly damaged proteins (12). HSPs such as HSP70, HSP27, and αβ-crystallin are constitutively expressed, but are also induced by stress in skeletal and cardiac muscles (13). Moreover, recent data revealed that plasma HSP72 is negatively correlated with muscle mass, weaker grip strength, and walking speed in men and women (14). However, although changes in HSPs with aging have been studied in blood (15,16), there is scant information regarding HSP expression in human aging and sarcopenia, and more crucially, potential relationships between HSPs and oxidative modifications. 

Therefore, despite the long-proposed links between aging, sarcopenia, and oxidative stress in skeletal muscle, relationships between protein oxidation, HSPs, and sarcopenia/muscle function (eg, strength) in old humans remain poorly defined. Our aim was to investigate possible differences, in young and old humans, of skeletal muscle protein carbonyl content and HSPs expression, and examine if these factors are linked to aging and/or sarcopenia, in cohorts of physiologically characterized humans.

## Materials and Methods

### Ethical Approval

Ethical approval was obtained from the University of Nottingham (D/2/2006), and the study was carried out in accordance with the Declaration of Helsinki. All volunteers were health-screened by a medical practitioner and provided written consent.

### Subject Characteristics and Study Design

Sixteen young and 16 old males not engaged in any exercise regime were recruited. One week prior to study, strength was assessed by 1-repetition maximum (1 RM) of the dominant leg on a dynamic leg extension machine (ISO leg extension, Leisure Lines [GB] Ltd) and body composition by dual-energy x-ray absorptiometry (GE Lunar Prodigy II, GE Healthcare). Following a general low load warm-up, participants began the 1 RM assessment by performing the leg extension at an intensity of approximately 50% of the estimated 1 RM. After 3-minute rest, the load was progressively increased in order to achieve the 1 RM in less than five attempts with 3-minute rest between each repetition. If it was not possible, the test was declared void and repeated 48 hours later. A subgroup of older men were classified as sarcopenic grade I according to the skeletal muscle index: appendicular lean mass/height^2^, when scoring >1 *SD* below that of the young group (17). This created three groups: young (Y), old (O), and old sarcopenic (OS, Table 1). Participants refrained from heavy activity 72 hour prior to study and fasted overnight before the study (water ad libitum). The next day (~08:30), musculus vastus lateralis biopsies were taken using conchotomes (18) under local anesthesia (1% lidocaine). Visible connective tissue/fat was removed before muscle was snap-frozen in liquid nitrogen and stored at −80°C prior to analysis.

**Table 1.** Participant Characteristics

	Young (*n* = 16)	Old (*n* = 8)	*p*	Old Sarcopenic (*n* = 8)	*p*
Age (y)	24±3	68±2		71±3	
Weight (kg)	81±10	79±10	ns	70±10	ns
Height (m)	1.82±0.04	1.77±0.03	ns	1.71±0.1^*,†^	.0001
BMI (kg/m^2^)	24±3	25±2	ns	24±2	ns
1 RM (kg)	81±14	43±13^*^	.0001	37±11^*^	.0001
Dominant lean leg mass (kg)	11.26±1563	9.86±952^*^	.0001	8.21±814^*,†^	.0001
ASM (kg)	30±4.6	26.7±1.9	ns	22.4±2.1^*^	.0001
SMI (kg/m^2^)	9.1±1.2	8.5±0.4	ns	7.6±0.3^*^	.001

*Notes:* ASM = appendicular skeletal muscle mass; BMI = body mass index; ns = nonsignificant; 1 RM = 1-repetition maximum; SMI = skeletal muscle index. Values are means ± *SD*.

^*^Different from young (Y) subjects.

^†^Different from old (O) subjects.

### Muscle Protein Subfraction (Myofibrillar, Sarcoplasmic, Mitochondrial) Isolations

Muscle (~25mg for mitochondrial and 15 mg for myofibrillar fractions) was separately homogenized in 10 μL·mg^−^^1^ of ice-cold buffer pH 7.5 (50 mM Tris–HCl, 1 mM EGTA, 1 mM EDTA, 10mM B-glycerophosphate, 50 mM NaF; Sigma-Aldrich, Poole, UK) and centrifuged at 10,000*g*. The supernatant (sarcoplasmic fraction) was removed and the myofibrillar pellet re-suspended (10 v/w) in ice-cold mitochondrial extraction buffer pH 7.5 (20 mM MOPS, 110 mM KCl, 1 mM EGTA) containing 0.25 mg·mL^−^^1^ of trypsin and incubated for 20 minutes on ice. Albumin was added to quench the reaction. Samples were spun for 5 seconds and the pellet washed and then homogenized in eight volumes of ice-cold mitochondrial extraction buffer without trypsin. Following this, samples were centrifuged for 5 minutes at 600*g* to pellet remaining myofibrils, and the supernatant was centrifuged for 10 minutes at 11,000*g* with the resulting pellet representing intramyofibrillar mitochondria. Protein concentrations were determined on a NanoDrop spectrophotometer (N0-1000V.3.8; Thermo Fisher Scientific). Fraction purities were determined by western blotting (see Western Blot Analysis for HSPs). Approximately, 10 μg of sarcoplasmic and myofibrillar protein fractions and 30 μg of mitochondrial protein fraction were electrophoresed and then ascertained using antibodies against eEF-2 (sarcoplasmic marker, 1:1,000; New England Biolabs, Hertfordshire, UK) and cytochrome C (mitochondrial marker, 1:1,000; Cell Signaling Technology, Inc., Beverly, MA). Loading was confirmed for each fraction using pan actin loading controls as stated below in the Western Blot Analysis for HSPs section and confirmed via Coomassie staining. Myofibrils are insoluble under these conditions (this fraction is typically used for MHC electrophoresis), but it was necessary to ensure that this fraction was not contaminated by mitochondria or cytoplasm.

### Western Blot Analysis for HSPs

Western blots were performed similarly as previously described (19). Briefly, 15 μg of sarcoplasmic and myofibrillar protein per sample was electrophoresed on 26-lane 12% sodium dodecyl sulfate–polyacrylamide gel electrophoresis gels (Criterion XT Bis-Tris; Bio-Rad, Hemel Hempstead, UK) before transfer to 0.2-μm polyvinylidene fluoride membranes on ice at 100V for 45 minutes. Membranes were blocked in 2.5% (w/v) skimmed milk in TBST (Tris-buffered saline and 0.1% Tween-20; both Sigma-Aldrich, Poole, UK) for 1 hour, prior to incubation in primary antibodies: goat polyclonal anti-Hsp27 (1:2,000; Santa Cruz Biotechnology, Santa Cruz, CA); mouse monoclonal anti-Hsp70 (1:1,000; Stressgen, Florence, Italy); mouse monoclonal anti-αβ-crystallin (1:1,000; Stressgen); and rabbit anti-pan-actin (1:2000; Cell Signaling Technology) overnight at 4°C. The next day, membranes were washed (3×) in TBST and incubated with horseradish peroxidase-linked secondary antibody for 1 hour at ambient temperature (1:2000; New England Biolabs). Membranes were developed using *electrochemiluminescence* for 5 minutes (Millipore Corp., Billerica, MA) and bands visualized/quantified by Chemidoc XRS (Bio-Rad Laboratories, Inc. Hercules, CA) ensuring no overexposure. Pan actin was used as a “loading control.”

### Oxyblot procedures to Determine Protein Carbonylation

The presence of protein carbonyl groups was assessed using the Oxyblot protein oxidation detection kit (Millipore) according to the manufacturer’s protocol. Briefly, the carbonyl groups in the protein side chains were derivatized to 2,4-dinitrophenylhydrazone by reaction with 2,4-dinitrophenylhydrazine. Precisely, 10 μg of protein was used for each sample, and the 2,4-dinitrophenol-derivatized protein samples were separated by polyacrylamide gel electrophoresis as described previously in the Western Blot Analysis for HSPs section. Polyvinylidene fluoride membranes were incubated for 1 hour in the stock primary antibody (1:150 in 1% PBS/TBST buffer), and after washing, for 1 hour in the stock secondary antibody (1:300 in % PBS/TBST buffer). Membranes were washed 3× in TBST and visualized as described previously in the Western Blot Analysis for HSPs section. The abundance of protein carbonylation was assessed by densitometry of each lane on a Chemidoc XRS system (Bio-Rad Laboratories, Inc.), again carefully ensuring avoidance of pixel saturation. Normalization for lane protein loading was performed using Coomassie staining.

### Statistical Analysis

Group differences were tested by one-way analysis of variance with Bonferroni post hoc testing. Correlations were assessed by Pearson’s product moment correlation coefficient. The alpha-level of significance was set at *p* ≤ .05 and two-tailed data are presented, except where indicated. Analyses were performed using GraphPad Prism version 5.0 (GraphPad software, La Jolla, CA). Data are mean ± *SEM* or means ± *SD*, as indicated.

## Results

### Subject Characteristics

The characteristics of participants are summarized in Table 1. Both 1 RM strength-related performance and lean leg mass by dual-energy x-ray absorptiometry were greater in the Y, compared with the O and OS groups. The O group had significantly greater leg muscle mass compared with the OS but similar strength levels.

### Purity of Subcellular Fractionation

**Figure 1.**
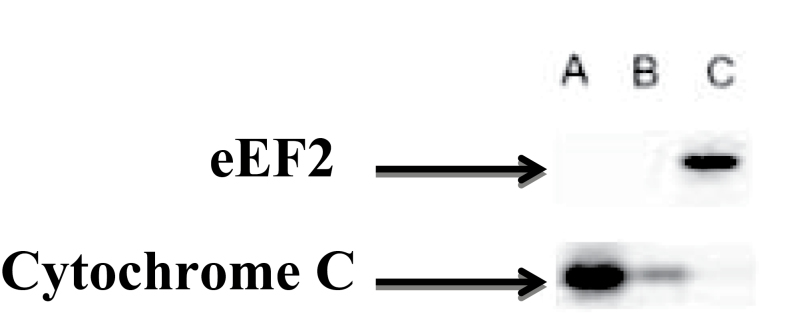
Purity of protein subfractions. Immunoblot representing the relative purity of mitochondrial (A), myofibrillar (B), and sarcoplasmic (C) preparations.

The method used for mitochondrial isolation has been validated previously (20). However, we have also provided confirmatory validation here (Figure 1), and as can be seen, the purity of our mitochondrial and sarcoplasmic fractions is acceptable. As described, myofibrils (action–myosin complexes) are insoluble in lysing buffers used for initial fractionation of sarcoplasmic fractions, and as can be seen (Figure 1), there is no mitochondrial contamination of the sarcoplasmic fraction. However, due to the presence of intramyofibrillar mitochondria, myofibrillar isolation may not always be free of mitochondria; nonetheless, any remaining mitochondrial contamination in our myofibrillar fractions was observed to be low versus our mitochondrial isolation (Figure 1).

### Protein Carbonyl Adducts

**Figure 2.**
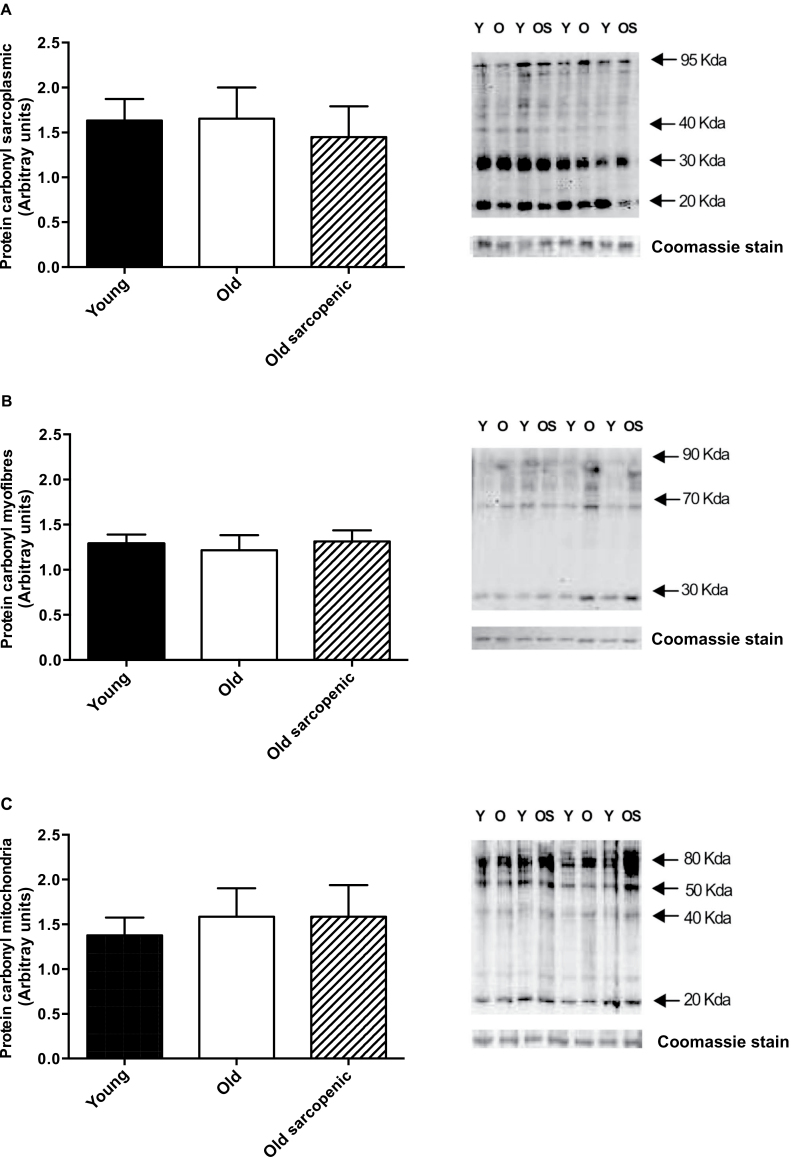
Immunochemical detection of protein carbonyls from vastus lateralis muscle of young (Y), old (O), and old sarcopenic (OS) groups: (**A**) sarcoplasmic fraction, (**B**) myofibrillar fraction, and (**C**) mitochondrial fraction. Data are measured in arbitrary units, measured as the ratio between the optical density (OD) obtained from the whole lane of the protein and the OD of Coomassie stain. Representative samples are shown. Values are presented as means ± *SEM*.

**Figure 3.**
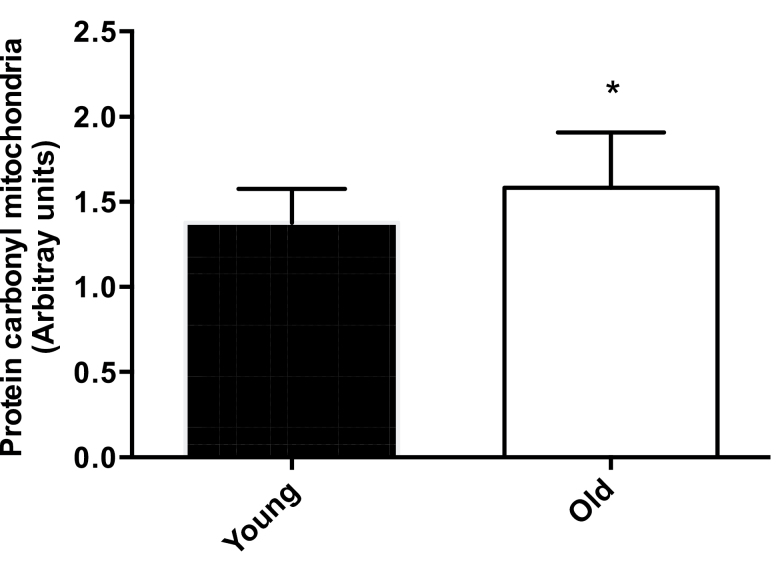
Mitochondrial carbonylation. Grouped old (O) + old sarcopenic (OS) versus young (Y) protein carbonylation data from the mitochondrial fractions. Values are presented as means ± *SEM*; *Indicates *p ≤* .05.

The abundance of protein carbonyl adducts was not different between Y, O, and OS groups for sarcoplasmic and myofibrillar fractions (Figure 2A and B). However, there was a trend toward increased formation in the mitochondrial fraction in the O and OS groups compared with Y (*p* = .08, Figure 2C). Interestingly, when older groups were combined, there was significantly greater (*p* = .03) “age-related” levels of protein carbonyl adducts present in the mitochondrial fraction in the O compared with the Y group (Figure 3).

### Correlation Between Protein Carbonyl Adducts, Muscle Mass, and Strength

**Figure 4.**
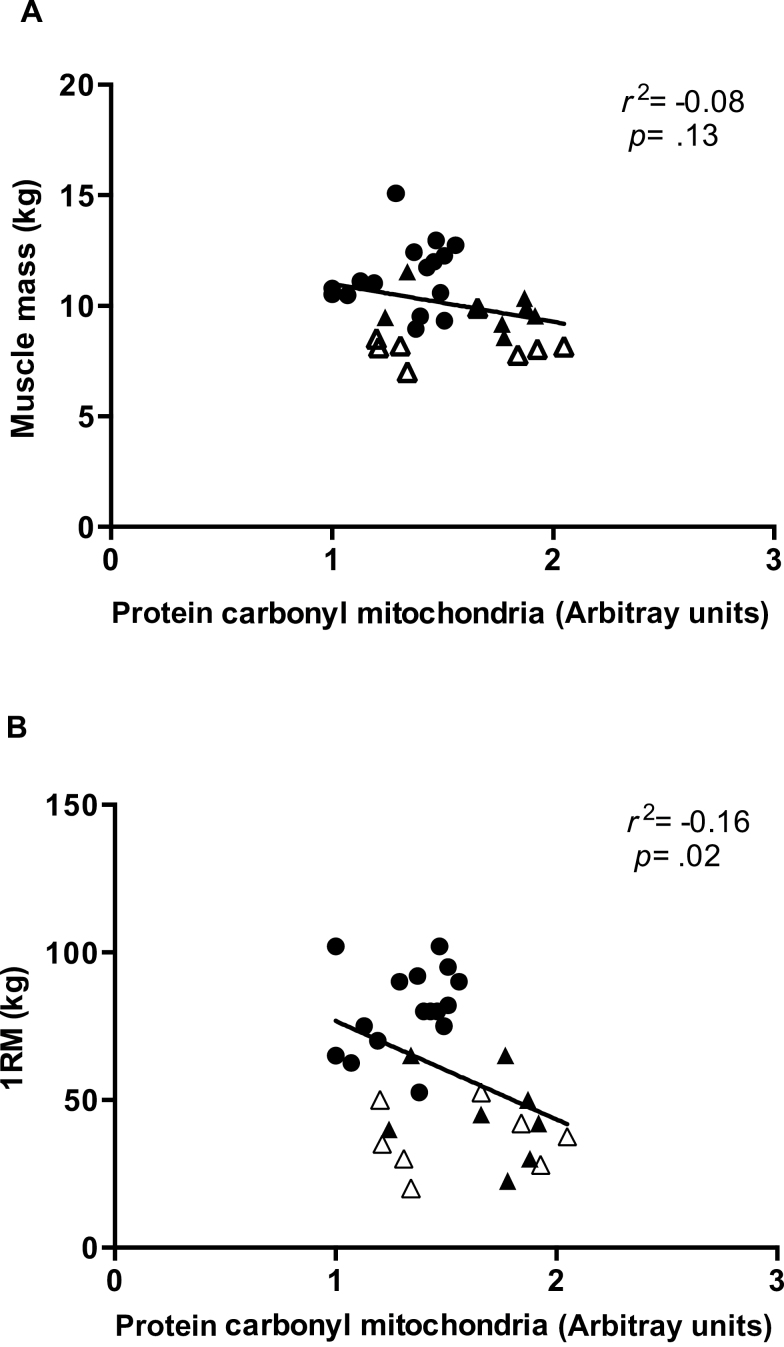
Correlation analysis between mitochondrial protein carbonyls and muscle mass (**A**) and strength (**B**). Circles indicate young (Y) subjects, filled triangles old (O) subjects, and empty triangles represent old sarcopenic (OS) subjects.

Protein carbonyl adducts from the myofibrillar, mitochondrial, or sarcoplasmic fractions were not correlated to muscle mass (Y: *p* = .45; O: *p* = .8; OS: *p* = .4) or strength (Y: *p* = .73; O: *p* = .36; OS: *p* = .9) when plotting each group individually (data not shown). However, when combining all subjects (32 subjects), a significant negative correlation was observed between protein carbonyl adducts from mitochondrial fraction and muscle strength (*p* = .02; two-tailed, *p* = .01; one-tailed, *r* ^2^ = −.16; Figure 4B), but not significantly with muscle mass (*p* = .13; two-tailed, *p* = .06; one-tailed, *r* ^2^ = −.08; Figure 4A). No such correlations were evident for the sarcoplasmic and myofibrillar fractions.

### HSP27, HSP70, αβ-Crystallin Protein Abundance

**Figure 5.**
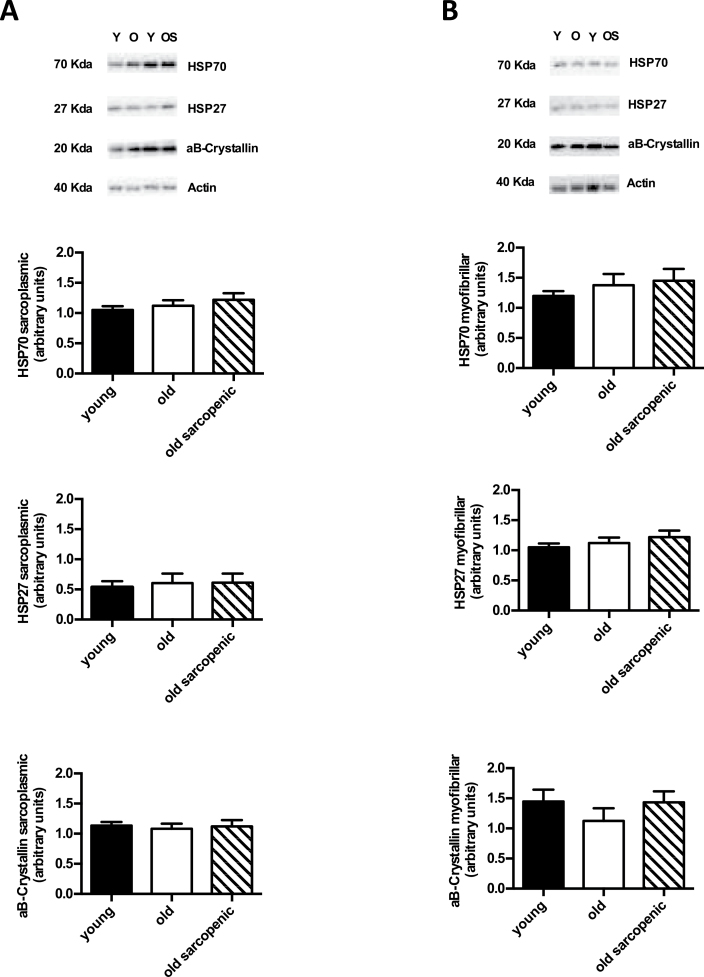
Immunochemical detection of heat shock protein (HSP) expression in muscle of young, old, and old sarcopenic subjects at rest. (**A**) HSPs analyzed in sarcoplasmic protein fraction and (**B**) HSPs from myofibrillar protein fraction. Data are measured in arbitrary units, measured as the ratio between the optical density (OD) of marker protein and the OD of pan actin. Representative samples are shown. Values are presented as means ± *SEM*.

For sarcoplasmic HSP70, HSP27, and αβ-crystallin, there were no group differences (Figure 5A). Similarly, there were no significant group differences in the myofibrillar protein fraction for the expression of HSP70, HSP27, and αβ-crystallin (Figure 5B).

## Discussion

To the best of our knowledge, this is the first study to investigate the abundance of key surrogate markers of oxidative damage: protein carbonyl adducts and HSPs, in multiple muscle fractions (myofibrillar, mitochondrial, and sarcoplasmic) and in groups of young and older humans. Moreover, from our cohort, we were uniquely able to isolate a group of older individuals designated as sarcopenic grade I, according to established criteria (17), enabling us to study the effects of aging in the absence and presence of sarcopenia. Our results reveal aging to be associated with increased abundance of protein carbonyl adducts, specifically in mitochondrial fractions. However, this increase in mitochondrial protein carbonylation was independent of sarcopenia at stage-I (ie, protein carbonyls were indistinguishable between O and OS). Furthermore, despite OS adults exhibiting reduced muscle strength and mass compared with Y, and lower muscle mass compared with O adults, OS individuals did not present increased mitochondrial protein carbonyls (vs O), or increased expression of HSP27, HSP70, and αβ-crystallin versus Y or O. Intriguingly, we observed a negative correlation between the degree of mitochondrial protein carbonylation and muscle strength (although allied correlations were not significant for muscle mass), independent of age and sarcopenia, per se.

This study provides new information relating to protein carbonylation in distinct muscle protein fractions and in relation to aging and sarcopenia. Indeed, to date, whether the abundance of protein carbonyls alters with aging has yielded incongruous findings (in immunoblots typically performed on the sarcoplasmic protein fraction). In our study, we report that neither sarcoplasmic nor myofibrillar abundance of protein carbonyls differed between Y, O, and OS groups. These results are in agreement with those of Marzani and colleagues (21) who reported similar protein carbonyl content between healthy young and older adults in both human vastus lateralis and rectus abdominis. However, they did report increased lipid peroxidation in the vastus lateralis of the older group. The 2,4-dinitrophenylhydrazine assay used to quantify protein carbonyls is considered reliable to detect total protein carbonyl formed from different reactions. Among those, lipid peroxidation products (like hydroxyl or malondialdehyde) may cause protein oxidation, although because these reactions are related to peroxidation of polyunsaturated membrane lipids, they may not always de facto, induce protein damage. Similarly, Mecocci and colleagues (22) and Pansarasa and colleagues (23) did not observe differences in protein carbonyl content between groups of patients undergoing orthopedic surgery. However, they reported a tendency for increased concentrations of protein carbonyls in old versus young muscle. In contrast, Gianni and colleagues (24) reported higher content (on average 170%) of sarcoplasmic protein carbonyls in vastus lateralis in old compared with younger men.

We next reasoned that oxidation of subcellular fractions (eg, mitochondria, myofibrils) or the presence or absence of sarcopenia could influence data gathered on a more “superficial” young versus old basis. For example, the rate of peroxide production by mitochondria reportedly increases with aging and has been inversely related to longevity (25). As a consequence, accumulation of damaged proteins due to oxidative stress may induce oxidative damage to mitochondrial DNA, impairing mitochondrial biogenesis and inducing apoptosis during aging (26). Indeed, recent evidence has further highlighted decreased mitochondrial function with aging. For instance, mitochondria isolated from the vastus lateralis muscle of older adults, exhibited lower adenosine triphosphate synthesis rates compared with young adults, indicative of decreased mitochondrial function with aging (27). In keeping with these findings, the authors also observed a reduced level of ROS production, along with no increase in lipid peroxidation (27). These data contrast the commonly held ROS theory of aging and suggest mitochondrial dysfunction with aging to be independent of oxidative stress. Moreover, this further highlights the lack of consensus within the literature with regards to the contribution of ROS production to aging and the need for further investigation of this phenomenon, directly in humans. In this study, we observed a tendency for increased protein carbonyl content in mitochondrial fractions when the O and OS groups were grouped, compared with the younger group (ie, an “age” effect). Although this may be linked to a role for oxidative stress in the impairment of mitochondrial function associated with aging (28), how this occurs in the absence of increased mitochondrial ROS production is unknown, though we speculate that this is due to the slower turnover of mitochondria with advancing age (29). Nevertheless, our results do imply that oxidized mitochondrial proteins in skeletal muscle of older adults may accumulate, independently of sarcopenia, at least for stage-I. Finally, as we observed a rather specific effect of aging on mitochondrial proteins, the lack of a consensus on the oxidation status of muscle proteins in aging may simply relate to cross-contamination of the fractions, thus highlighting the importance of assaying subfractions separately.

HSPs function as molecular chaperones, recognizing modified or mis-folded proteins, in order to ensure correct folding and protein repair under cellular stress (30). We show herein that HSP70, HSP27, and αβ-crystallin are not different between groups, that is, Y, O, and OS, in any of the protein fractions. Because HSPs are involved in the protein repair system (31), the unmodified expression of HSPs in muscle from young and older subjects supports our findings that there was no greater accumulation of damaged proteins in sarcoplasmic or myofibrillar fractions, though this in contrast to the modest age-dependent increases in the mitochondrial fraction. Previous investigations into age-related changes in the expression of HSPs in human skeletal muscle have been limited. In agreement with our data, Yamaguchi and colleagues (33) did not locate any difference in the expression of HSP27, αβ-crystallin, and HSP70 in young versus older subjects. Conversely Thalacker-Mercer and colleagues (33) reported increased expression of HSP70 in aged versus young human muscle, while in contrast, HSP27 was not expressed differently. Although the chaperone activities of HSPs have been suggested to decrease with age (34), our results indicate that human aging in either the absence or presence of designated sarcopenia is not associated with modulation of HSP abundance, per se (at least for those we measured), in leg skeletal muscle.

In conclusion, we have shown that protein carbonyl adducts in skeletal muscle were not altered in older subjects with sarcopenia (stage-I) versus those without. From this result, we conclude that protein carbonylation may not be directly responsible for the occurrence of sarcopenia. Nevertheless, as protein oxidation is an indirect measure of oxidative stress, we cannot discern links between ROS/reactive nitrogen species (RNS) and protein carbonylation in our subjects. Interestingly, increased abundance of mitochondrial protein carbonyls in older versus younger adults (older individuals were generally weaker and with reduced lean mass [see Table 1]), and the association between increased mitochondrial protein carbonylation and decreased muscle strength, points to mitochondrial oxidation status representing a previously undefined generic “marker” of low muscle strength. Finally, although there was a tendency toward an association between mitochondrial protein carbonylation and muscle mass (*p* = .13; two-tailed: *p* = .06; one-tailed: *r* ^2^ = −.08), further research with larger cohorts would be needed to confirm or refute such a relationship.

## Funding

The samples used within this study were from a grant supported by the Biotechnology and Biological Sciences Research Council (BB/X510697/1338 and BB/C516779/1).

## Conflict of Interest

The authors declare no conflicts of interest.
